# Association of Unhealthy Lifestyle and Genetic Risk Factors With Mild Cognitive Impairment in Chinese Older Adults

**DOI:** 10.1001/jamanetworkopen.2023.24031

**Published:** 2023-07-18

**Authors:** Huilian Duan, Dezheng Zhou, Ning Xu, Tong Yang, Qi Wu, Zehao Wang, Yue Sun, Zhenshu Li, Wen Li, Fei Ma, Yongjie Chen, Yue Du, Meilin Zhang, Jing Yan, Changqing Sun, Guangshun Wang, Guowei Huang

**Affiliations:** 1 Department of Nutrition and Food Science, School of Public Health, Tianjin Medical University, Tianjin, China; 2 Department of Epidemiology and Biostatistics, School of Public Health, Tianjin Medical University, Tianjin, China; 3 Department of Social Medicine and Health Management, School of Public Health, Tianjin Medical University, Tianjin, China; 4 Neurosurgical Department of Baodi Clinical College of Tianjin Medical University, Tianjin, China; 5 Department of Tumor, Baodi Clinical College of Tianjin Medical University, Tianjin, China; 6 Tianjin Key Laboratory of Environment, Nutrition and Public Health, Tianjin, China; 7 Department of Endocrinology and Metabolism, Tianjin Medical University General Hospital, Tianjin, China

## Abstract

**Importance:**

Apolipoprotein E polymorphism ε4 (*APOE* ε4) and methylenetetrahydrofolate reductase (*MTHFR*) TT genotype are genetic risk factors of mild cognitive impairment (MCI), but whether this risk can be changed by modifiable lifestyle factors is unknown.

**Objective:**

To explore whether unhealthy lifestyle (unhealthy dietary intake, current smoking, nonlimited alcohol consumption, and irregular physical activities) is associated with a higher risk of age-related MCI considering genetic risk.

**Design, Setting, and Participants:**

This population-based cohort study used data from Tianjin Elderly Nutrition and Cognition (TENC) study participants, recruited from March 1, 2018, through June 30, 2021, and followed up until November 30, 2022. Participants were Chinese adults aged 60 years or older who completed the neuropsychological assessments, general physical examinations, and a personal interview.

**Exposures:**

Healthy lifestyle was defined according to the Chinese Dietary Guidelines 2022, including healthy diet, regular physical activity, limited alcohol consumption, and no current smoking, categorized into healthy and unhealthy lifestyles according to weighted standardized lifestyle score. Genetic risk was defined by *MTHFR* TT genotype and *APOE* ε4, categorized into low and high genetic risk according to weighted standardized genetic risk score.

**Main Outcomes and Measures:**

The main outcome was newly diagnosed MCI as identified using a modified version of Petersen criteria. Hazard ratios (HRs) and 95% CIs were estimated using Cox proportional hazard regression models.

**Results:**

A total of 4665 participants were included (mean [SD] age, 67.9 [4.9] years; 2546 female [54.6%] and 2119 male [45.4%]); 653 participants with new-onset MCI (mean [SD] age, 68.4 [5.4] years; 267 female [40.9%] and 386 male [59.1%]) were identified after a median follow-up of 3.11 years (range, 0.82-4.61 years). Individuals with a low genetic risk and an unhealthy lifestyle (HR, 3.01; 95% CI, 2.38-3.79), a high genetic risk and a healthy lifestyle (HR, 2.65; 95% CI, 2.03-3.44), and a high genetic risk and an unhealthy lifestyle (HR, 3.58; 95% CI, 2.73-4.69) had a higher risk of MCI compared with participants with a low genetic risk and a healthy lifestyle. There was a synergistic interaction between lifestyle categories and genetic risk (β = 3.58; 95% CI, 2.73-4.69).

**Conclusions and Relevance:**

In this cohort study of TENC participants, the findings show that unhealthy lifestyle and high genetic risk were significantly associated with a higher risk of MCI among Chinese older adults. Unhealthy lifestyle factors were associated with a higher risk of MCI regardless of genetic risk, and lifestyle and genetic risk had synergistic interactions. These findings could contribute to the development of dietary guidelines and the prevention of early-stage dementia.

## Key Points

**Question:**

Is lifestyle associated with genetic risk factors of mild cognitive impairment (MCI) in Chinese older adults?

**Findings:**

In this cohort study of 4665 Chinese adults aged 60 years or older, unhealthy lifestyle was associated with a higher risk of MCI, regardless of genetic risk. In addition, a significant synergistic interaction between lifestyle categories and genetic risk was found.

**Meaning:**

The study’s findings of an association of unhealthy lifestyle with higher MCI risk and synergistic interactions between lifestyle and genetic risk could provide direction for the prevention of early-stage dementia.

## Teaser

This cohort study explores whether lifestyle and genetic risk factors, and their interaction, are associated with mild cognitive impairment in Chinese older adults.

## Introduction

Mild cognitive impairment (MCI) is a transitional cognitive stage between normal aging and dementia.^1^ Both lifestyle and genetic factors can play a role in the development of MCI.^2,3,4^ Apolipoprotein E polymorphism ε4 (*APOE* ε4) and methylenetetrahydrofolate reductase (*MTHFR*) TT genotype are well-recognized genetic risk factors for MCI and dementia.^5,6,7^ The status of *APOE* ε4 can contribute to the development of MCI by amyloid accumulation,^8^ whereas *MTHFR* TT genotype can mediate the level of homocysteine and vitamin B_12_ and folate concentrations, contributing to the incidence of MCI.^9^

There is a wealth of evidence that individuals who have healthy diets, get regular physical activity, limit alcohol consumption, and do not smoke can reduce their risk of MCI.^10,11,12,13^ Some studies have combined lifestyle factors into healthy lifestyle scores to investigate their association with other diseases, such as dementia and cardiovascular disease.^14,15^ A possible interaction between an unhealthy lifestyle and genetic risk may be associated with impaired cognition, but most studies analyzed this association based on *APOE* ε4, and the results have been controversial^16,17^ because of limited study samples and designs and exclusion of other genetic risk factors. In addition, the outcome of most studies was dementia and cognitive function rather than MCI.

In the context of a rapidly aging Chinese population, the trend of cognitive impairment is of great interest, and identifying associated risk factors is important. In this study, we analyzed longitudinal associations of genetic risk and lifestyle with the incidence of MCI, and the interaction of lifestyle and genetic risk, within the context of the Tianjin Elderly Nutrition and Cognition (TENC) cohort study. Lifestyle was defined based on the Chinese Dietary Guidelines (CDG) 2022, including dietary intake, physical activity, smoking status, and alcohol consumption. The purpose of this study was to identify potential intervention targets for MCI and to provide evidence for the optimization of dietary guidelines.

## Methods

### Study Participants

The TENC cohort study (Chinese Clinical Trial Register identifier ChiCTR2000034348) is an ongoing population-based prospective study focusing on nutrition and cognitive health of older adults in rural areas of north China. Participants without traumatic brain injury or concussion, aged 60 years or older, capable of walking, and with proper vision and hearing to complete the neuropsychological assessments were recruited from the Baodi District of Tianjin, China, from March 1, 2018, through June 30, 2021. Applying a multistage cluster sampling approach, we randomly selected 3 communities in the Baodi District. A total of 7304 qualified individuals were identified from these communities. Those willing to participate received general physical examinations and a personal interview conducted by licensed physicians and trained interviewers, respectively. Follow-up was performed from March 1, 2019, through November 30, 2022. Of the qualified individuals, 6426 participated in the genotyping, and 1761 were excluded from this analysis based on the following criteria: a history of Parkinson disease (n = 10), Alzheimer disease (n = 6), stroke (n = 361), or MCI (n = 847) (eFigure in Supplement 1) and missing questionnaire data (n = 537) (eTable 1 in Supplement 1). The remaining 4665 participants were included in the analysis, and we identified 653 participants with new-onset MCI. Salient characteristics of individuals included and excluded from the current study were largely comparable. The study protocol was approved by the ethics committee of Tianjin Medical University, and all participants provided oral informed consent before participation. All of our procedures followed the Strengthening the Reporting of Observational Studies in Epidemiology (STROBE) reporting guideline.

### Definition of MCI

Mild cognitive impairment was diagnosed using a modified version of the Petersen criteria^18^: (1) subjective memory disorders over at least 6 months; (2) Mini-Mental State Examination score of 17 points or less for illiteracy, 20 points or less for primary school, and 24 points or less for secondary education and above^19^; (3) absence of dementia (*Diagnostic and Statistical Manual of Mental Disorders, Fourth Edition* criteria), Alzheimer disease (National Institute of Neurological Disorders and Stroke Alzheimer Disease and Related Disorders Association criteria), psychiatric disorders, cerebral damage, or other physical diseases resulting in cognitive impairment; (4) cognitive performance of 1.5 SDs below the age-corrected (and education-corrected, where available) norms in at least 1 test in the neuropsychological battery; and (5) little or no difficulty in daily life activities as measured by the Activities of Daily Living Scale (<26 points).^20^ Participants with newly diagnosed MCI had to meet these 5 criteria, and the diagnosis was based on expert consensus by a panel of physicians, neurologists, neuropsychologists, and psychiatrists.

### Classification of Genetic Risk

During the clinical examination, we collected participants’ fasting venous blood in EDTA tubes, and genomic DNA was extracted using the QIAamp DNA Mini Kit (Spark Jade Science Co, Ltd). Genotypes were determined via a custom Taqman single nucleotide polymorphism genotyping assay by sequencing rs429358 and rs7412 at exon 4 of the *APOE* gene and rs1801133 of the *MTHFR* gene with the technical support of Shanghai OE Biotech Co, Ltd.

According to the literature, both *APOE* ε4 and *MTHFR* TT genotype are genetic risk factors for the development of MCI and Alzheimer disease.^21,22,23,24^ Therefore, we assigned 0 or 1 for both genetic factors in this study. Unweighted genetic risk score was the sum of the scores of both factors, ranging from 0 to 2 points (with higher scores indicating higher genetic risk), and then categorized as low (0 points) and high (1-2 points) genetic risks. Weighted standardized genetic risk scores were calculated based on β coefficients of both genetic factors using Cox proportional hazards regression models adjusted for age, sex, education level, living alone, monthly income, hypertension, diabetes, and 4 lifestyle factors (dietary intake, physical activity, smoking, and alcohol consumption). Each binary genetic variable was multiplied by the β coefficients, summed, divided by the sum of the β coefficients, and multiplied by 2. Based on the classification of the unweighted genetic risk score, the weighted standardized score was divided into low and high genetic risk.

### Classification of Lifestyle

The CDG 2022, promulgated by the government of the People’s Republic of China, is a guideline revised and compiled by the Chinese Nutrition Society that includes dietary and lifestyle behaviors and aims to help people make healthy dietary choices and behavior changes.^25^ In this study, lifestyle factors were defined according to the CDG 2022, including dietary intake, physical activity, smoking status, and alcohol consumption. We used the food frequency questionnaire to gain information about dietary intake; a short version of the International Physical Activity Questionnaire to obtain information about physical activities; and asked the standard questions, “Are you a current smoker?” and “Do you drink alcohol? If so, how much do you usually drink per day?”

Dietary intake items included (1) 200 to 300 g of cereals per day; (2) 300 g or more of fresh vegetables per day; (3) 200 g or more of fresh fruits per day; (4) 1 or more 250-mL servings of milk per day; (5) eating fish twice per week or 300 to 500 g per week, 300 to 350 g of eggs per week, 300 to 500 g of livestock and poultry per week; (6) no more than 5 g of salt per day; and (7) 25 to 30 g of cooking oil per day. Healthy diet was defined as meeting at least 4 of these 7 criteria.^14^ Regular physical activity was defined as at least 150 minutes of moderate-intensity physical activity per week. Limited alcohol consumption was defined as less than 15 g per day. Smoking status was categorized as current and no current smoking.

We assigned 0 or 1 for each lifestyle factor. The unweighted healthy lifestyle score was the sum of the scores of 4 factors, ranging from 0 to 4 points (with higher scores indicating lower adherence to a healthy lifestyle). Unweighted lifestyle was categorized as healthy (2-4 healthy lifestyle factors) and unhealthy (0-1 healthy lifestyle factors) based on the distribution of unweighted lifestyle scores. Weighted standardized healthy lifestyle score was calculated based on β coefficients of each lifestyle factor in the Cox proportional hazards regression model with all 4 lifestyle factors and adjusted for age, sex, education, living alone, monthly income, hypertension, diabetes, and *APOE* and *MTHFR* genotypes. Each binary lifestyle variable was multiplied by the β coefficients, summed, divided by the sum of the β coefficients, and multiplied by 4. Based on the distribution of the unweighted lifestyle score, the weighted standardized lifestyle score was divided into healthy and unhealthy lifestyle.^14^

### Other Variables

A structured questionnaire was designed to collect the participants’ demographic characteristics, including age, sex, education level, living alone, monthly income, and medical history. During the clinical examination, participants’ height and weight were measured and their body mass index (BMI) calculated by dividing their weight in kilograms by their height in meters squared.

### Statistical Analysis

Participant baseline characteristics are expressed as mean (SD) for continuous variables, while categorical variables are shown as counts and percentages. The Kolmogorov-Smirnov normality test was used to test the cumulative frequency distribution of continuous variables. Cox proportional hazard regression was performed to analyze the longitudinal association of lifestyle categories, genetic risks, and the combination of genetic risks and lifestyle categories (4 categories with low genetic risk and healthy lifestyle as reference), and the interaction of lifestyle and genetic risk, with the incidence of MCI; these results are presented as hazard ratios (HRs) with 95% CIs. Four-way decomposition models were used to test whether the interaction found was a mix of both interaction and mediation.

The longitudinal association between the 4 lifestyle factors and the incidence of MCI was analyzed in sensitivity analyses. Risk of MCI with lifestyle factors and genetic risks was further analyzed in sensitivity analyses using the combinations of unweighted lifestyle categories and weighted genetic risks, weighted lifestyle categories and unweighted genetic risks, and unweighted lifestyle categories and unweighted genetic risks. Additionally, we added BMI to lifestyle to recalculate this association, considering that some studies counted BMI as a lifestyle factor.^16,26,27^ We considered a BMI of 18.5 to less than 24 as a healthy lifestyle factor according to the revised Asia-Pacific BMI criteria by the World Health Organization ^28,29^ and the National Health Commission of the People’s Republic of China.^27^ Furthermore, we analyzed the combination of genetic risk and lifestyle categories with MCI stratified by sex, age, education level, hypertension, and diabetes. We also evaluated the association of lifestyle score and genetic risk score, both weighted and unweighted, with MCI. A 2-sided *P* < .05 was considered statistically significant. All analyses were performed using SAS, version 9.4 statistical software (SAS Institute, Inc).

## Results

This study included 4665 participants (mean [SD] age, 67.9 [4.9] years, 2546 female [54.6%] and 2119 male [45.4%]), with 653 with new-onset MCI (mean [SD] age, 68.4 [5.4] years; 267 females [40.9%], 386 males [59.1%]) after a median follow-up of 3.11 years (range, 0.82-4.61 years). A total of 878 participants (18.8%) met at least 4 of the 7 healthy diet criteria. Other baseline characteristics of the study participants with and without MCI are shown in Table 1.

**Table 1.** Characteristics of the Study Participants

Characteristic	No. (%)		
	All participants (N = 4665)	Without MCI (n = 4012)	With MCI (n = 653)
Age, mean (SD), y	67.9 (4.9)	67.8 (4.9)	68.4 (5.4)
Sex			
Female	2546 (54.6)	2279 (56.8)	267 (40.9)
Male	2119 (45.4)	1733 (43.2)	386 (59.1)
Education level			
Elementary school or less	2477 (53.1)	2240 (55.8)	237 (36.3)
Junior high school	1113 (23.9)	869 (21.7)	244 (37.4)
Senior high school and above	1075 (23.0)	903 (22.5)	172 (26.3)
BMI, mean (SD)	25.9 (3.3)	25.9 (3.3)	25.8 (3.3)
Living alone	327 (7.0)	279 (7.0)	48 (7.4)
Monthly income ≥3000 RMB	1946 (41.7)	1691 (42.2)	255 (39.1)
Hypertension	3147 (67.5)	2657 (66.2)	490 (75.0)
Diabetes	1385 (29.7)	1175 (29.3)	210 (32.2)
Healthy lifestyle characteristics			
Healthy diet	878 (18.8)	787 (19.6)	91 (13.9)
Regular physical activity	2146 (46.0)	1915 (47.7)	231 (35.4)
Limited alcohol consumption	4079 (87.4)	3551 (88.5)	528 (80.9)
Noncurrent smoker	3437 (73.7)	3031 (75.6)	406 (62.2)
No. of healthy lifestyle factors			
0	170 (3.6)	119 (3.0)	51 (7.8)
1	666 (14.3)	530 (13.2)	136 (20.8)
2	1923 (41.2)	1625 (40.5)	298 (45.6)
3	1596 (34.2)	1448 (36.1)	148 (22.7)
4	310 (6.6)	290 (7.2)	20 (3.1)
Weighted genetic risks			
Low	3241 (69.5)	2846 (70.9)	395 (60.5)
High	1424 (30.5)	1166 (29.1)	258 (39.5)
Weighted lifestyle categories			
Healthy	2146 (46.0)	1915 (47.7)	231 (35.4)
Unhealthy	2519 (54.0)	2097 (52.3)	422 (64.6)

Abbreviations: BMI, body mass index (calculated as weight in kilograms divided by height in meters squared); MCI, mild cognitive impairment; RMB, Renminbi.

### Longitudinal Association Between Healthy Lifestyles and MCI

As shown in Table 2, having a higher number of healthy lifestyle factors was associated with a lower risk of developing MCI, with or without adjustment for sociodemographic characteristics and genetic risks (1 lifestyle factor: HR, 0.68 [95% CI, 0.49-0.93]; 2 lifestyle factors: HR, 0.64 [95% CI, 0.47-0.87]; 3 lifestyle factors: HR, 0.32 [95% CI, 0.23-0.45]; 4 lifestyle factors: HR, 0.23 [95% CI, 0.13-0.39]; *P* for trend <.001).

**Table 2.** Longitudinal Association Between Healthy Lifestyles and Mild Cognitive Impairment

No. of healthy lifestyle factors	Univariable model			Multivariable model 1^a^				Multivariable model 2^b^			
	HR (95% CI)	*P* value	*P* value for trend	HR (95% CI)	*P* value	*P* value for trend	HR (95% CI)		*P* value	*P* value for trend
0	1 [Reference]	NA	<.001	1 [Reference]	NA	<.001	1 [Reference]		NA	<.001
1	0.65 (0.47-0.89)	.008		0.67 (0.48-0.92)	.01		0.68 (0.49-0.93)		.02	
2	0.56 (0.41-0.75)	<.001		0.62 (0.46-0.85)	.003		0.64 (0.47-0.87)		.004	
3	0.29 (0.21-0.40)	<.001		0.32 (0.23-0.45)	<.001		0.32 (0.23-0.45)		<.001	
4	0.20 (0.12-0.33)	<.001		0.23 (0.13-0.39)	<.001		0.23 (0.13-0.39)		<.001	

Abbreviations: HR, hazard ratio; NA, not applicable.

^a^ Model 1 adjusted for sex, age, education level, body mass index, income, hypertension, diabetes, and living alone.

^b^ Model 2 additionally adjusted for weighted genetic risks based on model 1.

### Association of Lifestyle Categories and Genetic Risks With MCI

**Figure 1.**
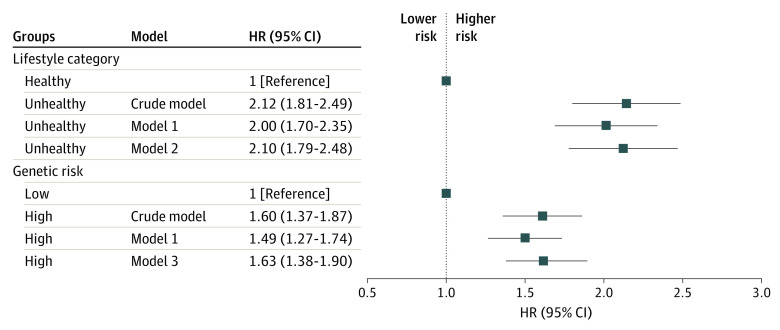
The Longitudinal Association Between Lifestyle Categories and Genetic Risks and Mild Cognitive Impairment Model 1 is adjusted for sex, age, education level, body mass index, income, hypertension, diabetes, and living alone. Model 2 is additionally adjusted for weighted genetic risks based on model 1. Model 3 is additionally adjusted for weighted lifestyle categories based on model 1. HR indicated hazard ratio.

Figure 1 shows that an unhealthy lifestyle was significantly associated with a higher risk of MCI in the univariable model (HR, 2.12; 95% CI, 1.81-2.49), adjusting for sociodemographic characteristics (HR, 2.00; 95% CI, 1.70-2.35) and additionally adjusting for genetic risks (HR, 2.10; 95% CI, 1.79-2.48). Individuals with high genetic risk had a higher risk of MCI in the univariable model (HR, 1.60; 95% CI, 1.37-1.87), adjusting for sociodemographic characteristics (HR, 1.49; 95% CI, 1.27-1.74) and additionally adjusting for lifestyle categories (high vs low: HR, 1.63; 95% CI, 1.38-1.90).

### Subgroup and Joint Effect Analysis

**Figure 2.**
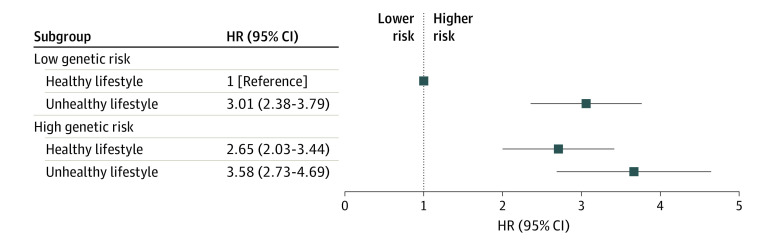
Risk of Mild Cognitive Impairment According to Lifestyle Categories and Genetic Risks Model adjusted for sex, age, education level, body mass index, income, hypertension, diabetes, and living alone. HR indicates hazard ratio.

Table 3 presents the risk of MCI according to lifestyle categories with genetic risks. Unhealthy lifestyle was significantly associated with a higher risk of MCI, regardless of low (HR, 2.96; 95% CI, 2.35-3.74) or high (HR, 1.38; 95% CI, 1.08-1.77) genetic risk. The association between MCI and the combination of lifestyle categories and genetic risks is shown in Figure 2. Participants with a low genetic risk and an unhealthy lifestyle (HR, 3.01; 95% CI, 2.38-3.79), a high genetic risk and a healthy lifestyle (HR, 2.65; 95% CI, 2.03-3.44), and a high genetic risk and an unhealthy lifestyle (HR, 3.58; 95% CI, 2.73-4.69) had a higher risk of MCI (*P* for trend <.001) compared with participants with a low genetic risk and a healthy lifestyle. A synergistic multiplicative interaction was observed between lifestyle categories and genetic risks (β = 3.58; 95% CI, 2.73-4.69), and this interaction was a mix of both interaction and mediation (mediated interaction, 0.12; 95% CI, 0.03-0.20; *P* = .006).

**Table 3.** Risk of MCI According to Lifestyle Categories With Genetic Risks^a^

Lifestyle	With MCI (n = 653)	Without MCI (n = 4012)	Hazard ratio (95% CI)	*P* value
Low genetic risk				
Healthy lifestyle	95	1336	1 [Reference]	<.001
Unhealthy lifestyle	300	1510	2.96 (2.35-3.74)	
High genetic risk				
Healthy lifestyle	136	579	1 [Reference]	.01
Unhealthy lifestyle	122	587	1.38 (1.08-1.77)	

Abbreviation: MCI, mild cognitive impairment.

^a^ Multivariable model adjusted for sex, age, education level, body mass index, income, hypertension, diabetes, and living alone.

### Sensitivity Analyses

In sensitivity analyses, we examined the longitudinal association between lifestyle factors and MCI, finding that unhealthy diet (HR, 1.31; 95% CI, 1.04-1.63), irregular physical activity (HR, 2.11; 95% CI, 1.79-2.48), and current smoking (HR, 1.25; 95% CI, 1.04-1.49) were associated with a higher risk of MCI (eTable 2 in Supplement 1). We also investigated various classifications to verify the associations of the combination of lifestyle categories and genetic risks with the risk of developing MCI, including (1) unweighted lifestyle categories and weighted genetic risks (eTable 3 in Supplement 1), (2) weighted lifestyle categories and unweighted genetic risks (eTable 4 in Supplement 1), (3) unweighted lifestyle categories and unweighted genetic risks (eTable 5 in Supplement 1), and (4) weighted lifestyle categories and weighted genetic risks with inclusion of BMI as a lifestyle factor (eTable 6 in Supplement 1). We stratified different sociodemographic factors for further validation to investigate their association with the results, including sex and age (eTable 7 in Supplement 1), education level (eTable 8 in Supplement 1), and prevalence of diabetes and hypertension (eTable 9 in Supplement 1). Furthermore, we analyzed the association of weighted and unweighted lifestyle scores and genetic risk scores with MCI and found higher scores to be associated with a higher risk of MCI (weighted lifestyle score: HR, 1.37; 95% CI, 1.29-1.47; weighted genetic risk score: HR, 1.51; 95% CI, 1.33-1.71) (eTable 10 in Supplement 1).

## Discussion

The findings of this population-based cohort study show that lifestyle and genetic risk were independently associated with risk of MCI. Unhealthy lifestyle was associated with a higher risk of MCI regardless of genetic risk. Study participants with a high genetic risk and an unhealthy lifestyle had a significantly higher risk of MCI than participants with a low genetic risk and a healthy lifestyle, and there were significant synergistic interactions between genetic risk and lifestyle.

To our knowledge, no studies have focused on the association between lifestyle and genetic risk (*APOE* and *MTHFR*) in the development of MCI, and most have focused on single lifestyle factors or the *APOE* gene instead of combined factors.^10,11,12,13,26^ A cross-sectional study of Chinese adults aged 80 years or older showed that healthy lifestyle was associated with better cognitive function in older adults, regardless of *APOE* genotype.^16^ Similarly, another longitudinal study from the UK Biobank reported that healthy lifestyle was associated with lower dementia risk among participants with high genetic risk, which was measured by a polygenetic risk score that included the *APOE* genotype.^14^ However, no significant interaction between genetic risk and lifestyle factors was found in either study, and the interaction was controversial. In a longitudinal study of Finnish adults, the authors stated that lifestyle interventions may modify dementia risk, particularly among individuals with high genetic risk.^30^ Yet, a prospective study of Finnish older adults reported that the risk of dementia increased with increasing alcohol consumption only in *APOE* ε4 carriers.^31^ Our results are consistent with these studies, and we found significant synergistic interactions, which were mixed with mediation, between genetic risk and lifestyle; that is, an unhealthy lifestyle is associated with an increased risk of MCI in older adults with high genetic risk, and genes could modulate the risk of MCI by regulating lifestyle factors. Since genetic factors cannot be changed, the risk of MCI could be reduced through lifestyle interventions. In particular, we defined lifestyle in this study according to the CDG 2022, so our results could provide scientific evidence to further optimize the dietary guidelines and lifestyle interventions.

Among the lifestyle factors, healthy diet and physical activity are of utmost importance, considering their greater HRs in this study. Unlike for other lifestyle factors, only 878 participants (18.8%) met at least 4 of the 7 healthy diet criteria. This percentage is quite low and consistent with a previous study from the China Health and Nutrition Survey.^32^ This phenomenon is a clear signal that more attention should be paid to diet-related knowledge of older adults living in rural areas and that dietary guidelines could be optimized for both urban and rural areas. Meanwhile, regular physical activity, no smoking, and limited alcohol consumption should be promoted vigorously to help to prevent MCI.

### Limitations

This study had several limitations. First, lifestyle factors were collected by questionnaire or standard questions and not randomly assigned as genetic factors. Second, lifestyle and genetic risk factors cannot be accurately classified despite standardization. Third, many other genes can also contribute to the development of MCI besides *APOE* ε4 and *MTHFR*; study of those genes can further improve the accuracy of genetic risk. Similarly, other lifestyle or external factors may also be associated with the incidence of MCI, such as environmental pollution. Fourth, the possibility for unmeasured confounders and reverse causality remains despite our adjustment for known potential factors. Fifth, the study sample comprised participants of the TENC cohort; therefore, further studies are warranted to determine the extent of extrapolation of these results to other populations. Furthermore, expanded sample size and an extended follow-up period are needed to further validate the current findings.

## Conclusions

In this cohort study, among older adult TENC participants without MCI, both unhealthy lifestyle and high genetic risk were significantly associated with a higher risk for MCI. Unhealthy lifestyle was associated with a higher risk of MCI among participants with both low and high genetic risk, and lifestyle and genetic risk had synergistic interactions. These findings could contribute to the development of dietary guidelines and interventions to prevent early-stage dementia.

## Notes

Supplement 1. **eFigure.** Flowchart of Study Participants **eTable 1.** The Frequency of Missing Variables **eTable 2.** The Longitudinal Association Between Lifestyle Factors and MCI **eTable 3.** Risk of MCI According to Unweighted Lifestyle Categories and Weighted Genetic Risks **eTable 4.** Risk of MCI According to Weighted Lifestyle Categories and Unweighted Genetic Risks **eTable 5.** Risk of MCI According to Unweighted Lifestyle Categories and Unweighted Genetic Risks **eTable 6.** Risk of MCI According to Weighted Lifestyle Categories and Weighted Genetic Risks **eTable 7.** Risk of MCI According to Lifestyle Categories and Genetic Risks Stratified by Sex and Age **eTable 8.** Risk of MCI According to Lifestyle Categories and Genetic Risks Stratified by Education Levels **eTable 9.** Risk of MCI According to Lifestyle Categories and Genetic Risks Stratified by Hypertension and Diabetes **eTable 10.** The Longitudinal Association Among Lifestyle Score, Genetic Risk Score, and MCI

Supplement 2.
Data Sharing Statement
