# Supplementary material for: Association of Unhealthy Lifestyle and Genetic Risk Factors With Mild Cognitive Impairment in Chinese Older Adults
Source: JAMA Netw Open. 2023 Jul 18;6(7):e2324031. doi: 10.1001/jamanetworkopen.2023.24031 (PMC10354670; doi:10.1001/jamanetworkopen.2023.24031)
Supplement: Supplement 1. — eFigure. Flowchart of Study Participants eTable 1. The Frequency of Missing Variables eTable 2. The Longitudinal Association Between Lifestyle Factors and MCI eTable 3. Risk of MCI According to Unweighted Lifestyle Categories and Weighted Genetic Risks eTable 4. Risk of MCI According to Weighted Lifestyle Categories and Unweighted Genetic Risks eTable 5. Risk of MCI According to Unweighted Lifestyle Categories and Unweighted Genetic Risks eTable 6. Risk of MCI According to Weighted Lifestyle Categories and Weighted Genetic Risks eTable 7. Risk of MCI According to Lifestyle Categories and Genetic Risks Stratified by Sex and Age eTable 8. Risk of MCI According to Lifestyle Categories and Genetic Risks Stratified by Education Levels eTable 9. Risk of MCI According to Lifestyle Categories and Genetic Risks Stratified by Hypertension and Diabetes eTable 10. The Longitudinal Association Among Lifestyle Score, Genetic Risk Score, and MCI [file jamanetwopen-e2324031-s001.pdf]

Supplemental Online Content

Duan H, Zhou D, Xu N, et al. Association of unhealthy lifestyle and genetic risk factors with mild cognitive impairment in Chinese older adults. *JAMA Netw Open*. 2023;6(7):e2324031. doi:10.1001/jamanetworkopen.2023.24031

eFigure. Flowchart of Study Participants

eTable 1. The Frequency of Missing Variables

eTable 2. The Longitudinal Association Between Lifestyle Factors and MCI

eTable 3. Risk of MCI According to Unweighted Lifestyle Categories and Weighted Genetic Risks

eTable 4. Risk of MCI According to Weighted Lifestyle Categories and Unweighted Genetic Risks

eTable 5. Risk of MCI According to Unweighted Lifestyle Categories and Unweighted Genetic Risks

eTable 6. Risk of MCI According to Weighted Lifestyle Categories and Weighted Genetic Risks

eTable 7. Risk of MCI According to Lifestyle Categories and Genetic Risks Stratified by Sex and Age

eTable 8. Risk of MCI According to Lifestyle Categories and Genetic Risks Stratified by Education Levels

eTable 9. Risk of MCI According to Lifestyle Categories and Genetic Risks Stratified by Hypertension and Diabetes

eTable 10. The Longitudinal Association Among Lifestyle Score, Genetic Risk Score, and MCI

This supplemental material has been provided by the authors to give readers additional information about their work.

© 2023 Duan H et al. *JAMA Network Open.*

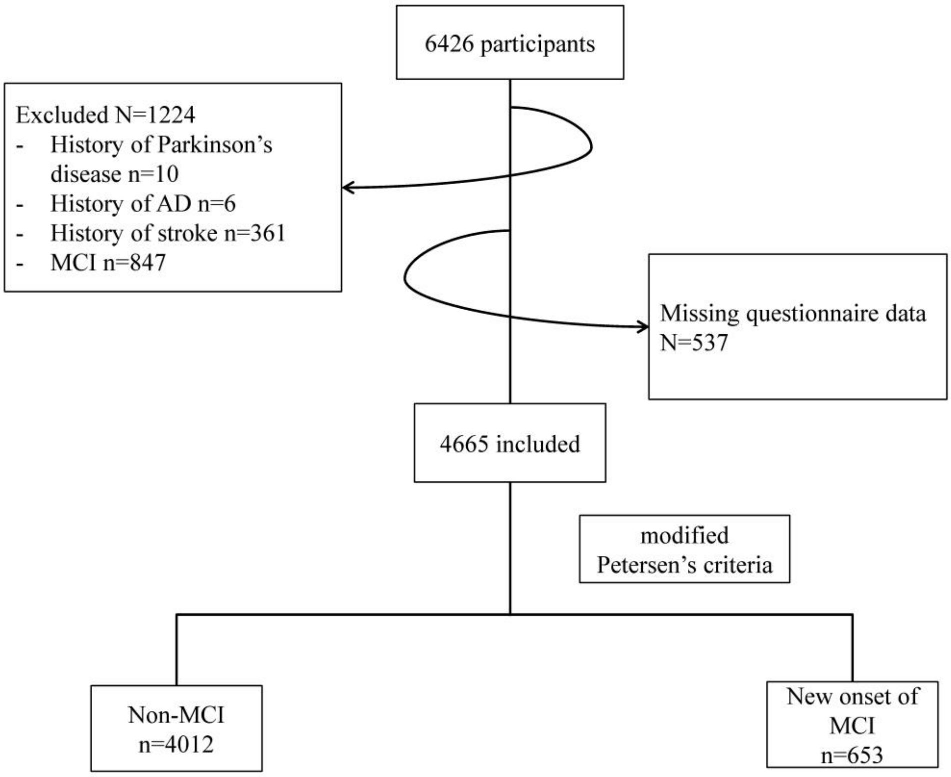

eFigure. Flowchart of Study Participants.

AD, Alzheimer's disease; MCI, mild cognitive impairment.

© 2023 Duan H et al. *JAMA Network Open.*

eTable 1. The Frequency of Missing Variables

Variables	Missing (%)
Body mass index	1.51
Baseline mild cognitive impairment information	0.12
Lost to follow-up	0.33
Smoking status	0.54
Alcohol consumption	0.82
Fruit intake	0.75
Salt intake	2.32
Cooking oil consumption	0.44
Cereal intake	1.04
Vegetable intake	3.38
Egg intake	0.17
Meat intake	3.31
Physical activities	1.12

© 2023 Duan H et al. *JAMA Network Open.*

eTable 2. The Longitudinal Association Between Lifestyle Factors and MCI

Lifestyle Characteristics	Univariate Model		Multivariable Model 1[a]		Multivariable Model 2[b]	
	***HR* (95% *CI*)**	***P* Value**	***HR* (95% *CI*)**	***P* Value**	***HR* (95% *CI*)**	***P* Value**
Healthy diet		.05		.01		.02
Yes	1 [Reference]		1 [Reference]		1 [Reference]	
No	1.24 (1.00-1.55)		1.34 (1.07-1.67)		1.31 (1.04-1.63)	
Regular physical activity		<.001		<.001		<.001
Yes	1 [Reference]		1 [Reference]		1 [Reference]	
No	2.12 (1.81-2.49)		2.00 (1.70-2.35)		2.11 (1.79-2.48)	
Limited alcohol drinking		<.001		.07		.23
Yes	1 [Reference]		1 [Reference]		1 [Reference]	
No	1.62 (1.33-1.97)		1.22 (0.98-1.51)		1.14 (0.92-1.42)	
Non-current smoking		<.001		.004		.02
Yes	1 [Reference]		1 [Reference]		1 [Reference]	
No	1.46 (1.24-1.70)		1.30 (1.09-1.55)		1.25 (1.04-1.49)	

[a] Multivariable model 1 adjusted for sex, age, education levels, BMI, income, hypertension, diabetes and living alone.

[b] Multivariable model 2 additionally adjusted weighted genetic risks and lifestyle factors (smoking status, alcohol consumption, physical activity and dietary intake) based on multivariable model 1.

© 2023 Duan H et al. *JAMA Network Open.*

eTable 3. Risk of MCI According to Unweighted Lifestyle Categories and Weighted Genetic Risks[a]

Subgroup	MCIs / Non-MCIs (653 / 4012)	*HR* (95% *CI*)	*P* Value	*P* for trend
Low genetic risk				<.001
Healthy lifestyle	62 / 1236	1 [Reference]		
Unhealthy lifestyle	333 / 1610	3.60 (2.73-4.74)	<.001	
High genetic risk				
Healthy lifestyle	106 / 502	3.34 (2.44-4.58)	<.001	
Unhealthy lifestyle	152 / 664	4.16 (3.08-5.62)	<.001	

[a] Model adjusted for sex, age, education levels, BMI, income, hypertension, diabetes and living alone.

eTable 4. Risk of MCI According to Weighted Lifestyle Categories and Unweighted Genetic Risks[a]

Subgroup	MCIs / Non-MCIs (653 / 4012)	*HR* (95% *CI*)	*P* Value	*P* for trend
Low genetic risk				<.001
Healthy lifestyle	49 / 1146	1 [Reference]		
Unhealthy lifestyle	259 / 1316	4.78 (3.52-6.49)	<.001	
High genetic risk				
Healthy lifestyle	182 / 769	4.39 (3.20-6.02)	<.001	
Unhealthy lifestyle	163 / 781	5.68 (4.12-7.83)	<.001	

[a] Model adjusted for sex, age, education levels, BMI, income, hypertension, diabetes and living alone.

© 2023 Duan H et al. *JAMA Network Open.*

eTable 5. Risk of MCI According to Unweighted Lifestyle Categories and Unweighted Genetic Risks[a]

Subgroup	MCIs / Non-MCIs (653 / 4012)	*HR* (95% *CI*)	*P* Value	*P* for trend
Low genetic risk				<.001
Healthy lifestyle	24 / 1062	1 [Reference]		
Unhealthy lifestyle	284 / 1400	7.33 (4.82-11.16)	<.001	
High genetic risk				
Healthy lifestyle	144 / 676	7.02 (4.56-10.82)	<.001	
Unhealthy lifestyle	201 / 874	8.96 (5.85-13.74)	<.001	

[a] Model adjusted for sex, age, education levels, BMI, income, hypertension, diabetes and living alone.

eTable 6. Risk of MCI According to Weighted Lifestyle Categories[a] and Weighted Genetic Risks[b]

Subgroup	MCIs / Non-MCIs (653 / 4012)	*HR* (95% *CI*)	*P* Value	*P* for trend
Low genetic risk				<.001
Healthy lifestyle	119 / 1565	1 [Reference]		
Unhealthy lifestyle	276 / 1281	2.79 (2.25- 3.47)	<.001	
High genetic risk				
Healthy lifestyle	151 / 668	2.41 (1.89-3.07)	<.001	
Unhealthy lifestyle	107 / 498	3.23 (2.48-4.20)	<.001	

[a] Healthy lifestyle including non-current smoking, limited alcohol drinking, regular physical activity, healthy diet and normal BMI. [b] Model adjusted for sex, age, education levels, income, hypertension, diabetes and living alone.

© 2023 Duan H et al. *JAMA Network Open.*

eTable 7. Risk of MCI According to Lifestyle Categories and Genetic Risks Stratified by Sex and Age

Subgroup	Male (n=2119) [a]		Female (n=2546) [a]		Age <70 (n=3042) [b]		age≥70 (n=1623) [b]	
	***HR* (95% *CI*)**	***P* Value**	***HR* (95% *CI*)**	***P* Value**	***HR* (95% *CI*)**	***P* Value**	***HR* (95% *CI*)**	***P* Value**
**Low genetic risk**								
Healthy lifestyle	1 [Reference]		1 [Reference]		1 [Reference]		1 [Reference]	
Unhealthy lifestyle	2.41 (1.81-3.22)	<.001	4.08 (2.76-6.02)	<.001	2.73 (2.06-3.63)	<.001	3.80 (2.53-5.71)	<.001
**High genetic risk**								
Healthy lifestyle	1.95 (1.38-2.77)	<.001	3.93 (2.60-5.96)	<.001	2.52 (1.82-3.48)	<.001	2.98 (1.88-4.71)	<.001
Unhealthy lifestyle	2.71 (1.92-3.84)	<.001	5.55 (3.58-8.59)	<.001	3.34 (2.38-4.71)	<.001	4.39 (2.79-6.90)	<.001
***P* for trend**	<.001		<.001		<.001		<.001	

[a] Model adjusted for age, education levels, BMI, income, hypertension, diabetes and living alone.

[b] Model adjusted for sex, education levels, BMI, income, hypertension, diabetes and living alone.

© 2023 Duan H et al. *JAMA Network Open.*

eTable 8. Risk of MCI According to Lifestyle Categories and Genetic Risks Stratified by Education Levels[a]

Subgroup	Elementary school or below (n=2477)			Junior high school and above (n=2188)		
	***HR* (95% *CI*)**	***P* Value**	***P* for trend**	***HR* (95% *CI*)**	***P* Value**	***P* for trend**
**Low genetic risk**			<.001			<.001
Healthy lifestyle	1 [Reference]			1 [Reference]		
Unhealthy lifestyle	6.05 (3.759-9.76)	<.001		2.24 (1.71-2.94)	<.001	
**High genetic risk**						
Healthy lifestyle	4.99 (2.96-8.40)	<.001		2.01 (1.47-2.74)	<.001	
Unhealthy lifestyle	7.70 (4.55-13.01)	<.001		2.52 (1.82-3.48)	<.001	

[a] Model adjusted for age, sex, BMI, income, hypertension, diabetes and living alone.

© 2023 Duan H et al. *JAMA Network Open.*

eTable 9. Risk of MCI According to Lifestyle Categories and Genetic Risks Stratified by Hypertension and Diabetes

Subgroup	Hypertension (n=3147) [a]		Non-hypertension (n=1518) [a]		Diabetes (n=1385) [b]		Non-diabetes (n=3280) [b]	
	***HR* (95% *CI*)**	***P* Value**	***HR* (95% *CI*)**	***P* Value**	***HR* (95% *CI*)**	***P* Value**	***HR* (95% *CI*)**	***P* Value**
**Low genetic risk**								
Healthy lifestyle	1 [Reference]		1 [Reference]		1 [Reference]		1 [Reference]	
Unhealthy lifestyle	2.89 (2.21-3.78)	<.001	3.20 (2.01-5.09)	<.001	2.71 (1.79-4.10)	<.001	3.23 (2.44-4.28)	<.001
**High genetic risk**								
Healthy lifestyle	2.53 (1.86-3.43)	<.001	2.92 (1.73-4.94)	<.001	2.26 (1.44-3.55)	<.001	2.85 (2.06-3.95)	<.001
Unhealthy lifestyle	3.27 (2.39-4.48)	<.001	4.15 (2.42-7.10)	<.001	3.19 (1.98-5.14)	<.001	3.80 (2.73-5.28)	<.001
***P* for trend**	<.001		<0.001		<0.001		<0.001	

[a] Model adjusted for age, sex, education levels, BMI, income, diabetes and living alone.

[b] Model adjusted for age, sex, education levels, BMI, income, hypertension and living alone.

© 2023 Duan H et al. *JAMA Network Open.*

eTable 10. The Longitudinal Association Among Lifestyle Score, Genetic Risk Score, and MCI[a]

Scores	Univariate Model		Multivariable Model	
	***HR* (95% *CI*)**	***P* Value**	***HR* (95% *CI*)**	***P* Value**
Weighted lifestyle score	1.42 (1.33-1.51)	<.001	1.37 (1.29-1.47)	<.001
Weighted genetic risk score	1.61 (1.42-1.83)	<.001	1.51 (1.33-1.71)	<.001
Unweighted lifestyle score	1.48 (1.37-1.61)	<.001	1.44 (1.32-1.58)	<.001
Unweighted genetic risk score	1.44 (1.30-1.59)	<.001	1.38 (1.24-1.52)	<.001

[a] Model adjusted for sex, age, education levels, BMI, income, hypertension, diabetes and living alone.

© 2023 Duan H et al. *JAMA Network Open.*
