# Supplementary material for: Association of Unhealthy Lifestyle and Genetic Risk Factors With Mild Cognitive Impairment in Chinese Older Adults
Source: JAMA Netw Open. 2023 Jul 18;6(7):e2324031. doi: 10.1001/jamanetworkopen.2023.24031 (PMC10354670; doi:10.1001/jamanetworkopen.2023.24031)
Supplement: Supplement 2. — Data Sharing Statement [file jamanetwopen-e2324031-s002.pdf]

Data Sharing Statement

Duan. Association of Unhealthy Lifestyle and Genetic Risk Factors With Mild Cognitive Impairment in Chinese Older Adults. *JAMA Netw Open*. Published July 18, 2023. doi:10.1001/jamanetworkopen.2023.24031

Data
Data available: No

Additional Information
Explanation for why data not available: This cohort study is in the process of being followed-up.
